# Analysis of mortality trends in children aged 0–14 in Brazil and the northeast macroregion: a time series study (2000–2019)

**DOI:** 10.3389/fped.2025.1649701

**Published:** 2025-11-12

**Authors:** Robenilson Diniz Alves, Cristiane da Silva Ramos Marinho, Janmilli da Costa Cantas Santiago, Yago Tavares Pinheiro, Osvaldo De Goes Bay Junior, Klayton Galante Sousa

**Affiliations:** 1 Faculdade de Ciências da Saúde do Trairi—FACISA, Programa de Pós-Graduação em Saúde Coletiva–PPGSacol, Universidade Federal do Rio Grande do Norte—UFRN, Santa Cruz, Brazil; 2 Graduate Program in Public Health, Federal University of Rio Grande do Norte, Santa Cruz, Rio Grande do Norte, Brazil; 3 Department of Nursing, Federal University of Rio Grande do Norte, Natal, Rio Grande do Norte, Brazil; 4 Department of Physiotherapy, Santa Maria University Center, Cajazeiras, Paraíba, Brazil

**Keywords:** child deaths, child health, public policy, causes of death, epidemiological studies, epidemiological surveillance

## Abstract

**Introduction:**

Child deaths in Brazil have fallen significantly in recent decades. However, mortality rates remain high compared to countries with a Human Development Index considered very high. In addition, social problems and inequalities remain evident and require the promotion of effective public policies. However, since the beginning of the millennium, a governmental effort in favor of children's health has been underway, with actions that have contributed to improving mortality rates. In this context, it is essential to investigate the transformations of the first two decades of the 21st century.

**Methods:**

This study aims to describe and identify the temporal trends in mortality rates for children aged 0–14 in Brazil and its macro-regions. This is an ecological, time-series study with a quantitative approach, based on secondary data on infant mortality (<1 year), childhood mortality (<5 years), and mortality in children between 5 and 14 years, in Brazil and in the Northeast Macroregion, between 2000 and 2019. Data was collected from January to August 2024 through the Mortality, Live Births, and Resident Population Information Systems provided by the Brazilian Institute of Geography and Statistics (IBGE). Trends were analyzed using *Joinpoint* statistical software.

**Results:**

In Brazil and the Northeast, there was a significant downward trend in infant and childhood mortality rates until 2014, after which the curve remained stationary. Among children aged 5–14, a continuous downward trend was observed, with regional and temporal variations, particularly a greater decrease after 2013 in the country and in 2012 in the Northeast. Black children had a higher percentage of deaths, males were more prevalent, and perinatal conditions were the main cause of death among children under 1 and 5 years old. External causes of morbidity and mortality prevailed as the main causes of death among children aged 5–14.

**Discussion:**

During the two decades studied, there was a significant reduction in mortality. However, the stagnation of rates since 2014 and the uneven profile of deaths indicate that social indicators and health actions were impacted by austerity measures, posing a challenge for the country in terms of maintaining, regionalizing, and strengthening effective public policies.

## 1 Introduction

Over the years, the vast regions of Brazil have been impacted by various factors that have influenced morbidity and mortality indicators in the child health scenario. Data from the United Nations (UN) show that the country achieved the global goal proposed by the Millennium Development Goals (MDGs) in 2000, by reducing the Infant Mortality Rate (IMR) by two-thirds in children under 1 year of age (1).

Between 1990 and 2015, the IMR in Brazil fell from 47.1 to 13.3 infant deaths per thousand live births (LB) (2). In 2022, the IMR in the country was 12.6 deaths per thousand live births (3). In the Northeast, the IMR went from 75.8 in 1990 to 15.2 per thousand LB in 2019 (2).

Mortality rates varied between Brazilian macro-regions. In 2022, estimates of the Infant Mortality Rate (IMR) revealed the Northeast with a rate of 16.7 per 1,000 LB; the North with 18.5 deaths per 1,000 LB; the Midwest with 15.3 deaths per 1,000 LB; the Southeast with 13.8 deaths per 1,000 LB and the South with 12.1 deaths per 1,000 LB (4).

Even with the implementation of public policies, mortality rates among children in different age groups are still considerably higher when compared to developed countries (5). According to the *Inter-Agency Group for Child Mortality Estimation* (UN IGME), in 2022, Brazil had an IMR of 2.53 per thousand LB, an IMRf of 14.02 per thousand LB, and a mortality rate for children aged 5–14 of 2.33 per thousand 5-year-olds. In comparison, in Canada, the IMR was 4.34 per thousand LB; the IMRf was 4.94 per thousand LB; and the mortality rate for children aged 5–14 was 0.88 per thousand 5-year-olds; in Norway, the IMR was 1.76 per thousand LB; the IMRf was 2.17 per thousand LB; the mortality rate for children aged 5–14 was 0.67 per thousand children aged 5; and in Japan, the IMR was 1.72 per thousand LB; the IMRin was 2.28 per thousand LB and the mortality rate for children aged 5–14 was 0.72 per thousand children aged 5, revealing significant disparities (6).

Among children and adolescents aged 5–14, the causes of death differ from those of the younger age groups. It is estimated that around 1 million children in this age group died worldwide in 2016, mainly from external causes (7). In Brazil, around 21% of girls and 32% of boys were killed by traffic accidents, drowning, and homicides (8).

Although there have been reductions in child mortality rates, this reduction has not occurred equally between social groups. Children belonging to lower-income families have a two to three times higher risk of death by the age of five (9, 10). In fact, according to the *World Inequality Lab* (2023), Brazil remains one of the countries with the highest levels of social and income inequality globally.

Territorial inequalities also have a significant impact on morbidity and mortality, primarily due to difficulties in access, such as geographical distance (11). In 2022, the North and Northeast regions had the most significant shares of the population without access to water and sewage collection, according to the Trata Brasil Institute (12), which contributes to maintaining negative indicators.

Faced with this scenario, Brazil adhered to the Sustainable Development Goals (SDGs), created by the UN in 2015, which set targets to be achieved by 2030. A global appeal that prioritizes 17 goals to promote equity. SDG 3—Health and Well-being, proposes, for example, in target 3.1, to reduce the global maternal mortality ratio to 70 deaths per 100,000 LB and in target 3.2, to end the number of preventable deaths of children aged 0–5, in addition to reducing neonatal mortality to 12 per thousand LB and in children under 5 to at least 25 per thousand LB (1).

In this approach to preventing child deaths, the spatial distribution of this segment must be investigated, given the link with inequalities in the territory (13). Target 3.4 aims to reduce premature death caused by non-communicable diseases to one third, with a view to prevention and treatment; promotion of mental health and well-being. Target 3.8 aims to achieve universal health coverage, with adequate essential services and the availability of essential medicines and vaccines (1).

Over the past two decades, critical mortality events have occurred, with a focus on socioeconomic and epidemiological factors. Given this, it is necessary to investigate the evolution of infant, childhood, and child mortality indicators between the ages of 5 and 14 in Brazil and the Northeast region of the country, between 2000 and 2019. Given the above, the question arises: What is the behavior of infant mortality rates (<1 year), childhood mortality rates (<5 years) and mortality rates for children aged 5–14 years in Brazil and in the Northeast Macroregion from 2000 to 2019? What are the characteristics of these deaths in the two decades analyzed?

Given this reality, it is essential to describe and identify temporal trends in mortality rates among the selected age groups, analyzing possible changes over time among the macro-regions, with an emphasis on social profile, causes of death, and changes in epidemiological characteristics.

## 2 Methods

This is a descriptive time-series ecological study with a quantitative approach, based on publicly available secondary data from the Department of Information Technology of the Unified Health System (DATASUS). The TabNet tool was used to tabulate the data, accessing the Mortality Information System—SIM, the Live Birth Information System—SINASC, as well as population data obtained from the Study of Population Estimates by Municipality, provided by the Brazilian Institute of Geography and Statistics (IBGE).

The use of indicators in descriptive studies is a widely employed strategy in epidemiology, enabling the utilization of secondary data, such as mortality data, or even primary data collected during the study itself. Time series analysis is an essential approach within this scope, allowing for the organization and interpretation of quantitative data over time, which can be applied to different types of research (14, 15).

For this research, Brazil and the Northeast Macroregion were used as units of analysis, with an investigation of specific indicators related to child mortality. The population composition of Brazil was based on the 2022 demographic census, which estimated 203,080,756 inhabitants, distributed among the 27 Federation Units, in 5,570 municipalities, divided into five regions of the country (North, Northeast, South, Southeast and Midwest). The population of the Northeast region was estimated at 54,658,515 inhabitants, distributed among the region's nine states: Alagoas (AL), Bahia (BA), Ceará (CE), Maranhão (MA), Paraíba (PB), Pernambuco (PE), Piauí (PI), Rio Grande do Norte (RN) and Sergipe (SE), with a population density of 35.21 inhabitants per km^2^. In 2022, a total of 40,129,261 children under the age of 14 were estimated in the country, with the North Macroregion, followed by the Northeast, having the youngest population (16).

The data was collected between January and August 2024. The data extracted from DATASUS refers to the period between 2000 and 2019, targeting children in the age groups: under 1 year old, under 5 years old, and 5–14 years old, both in Brazil and in the Northeast Macroregion. The data was organized into tables and exported into files in CSV format for later analysis.

The mortality coefficients were calculated for infants (under 1 year old), children (under 5 years old) and children aged 5–14, as well as the percentage of deaths according to race/color, sex and chapter of the International Classification of Diseases—10th revision (ICD-10), for the 0–14 age group.

The dependent variables were: Infant Mortality Coefficients (under 1 year), Child Mortality Coefficient (under 5 years) and the Mortality Coefficient for children aged 5–14 years. The independent variables were race/color, gender and age group, with data expressed in absolute and relative values. The initial calculations were carried out using *Microsoft Excel* 2013 software. Details of the variables and data sources are shown in Table 1.

**Table 1:** Operational definition of the variables used in the study.

Variable	Type	Operational definition	Data source
Infant mortality coefficient (CMI)	Dependent	Number of deaths in children under 1 year of age in a given locality divided by the total number of live births in a given locality, multiplied by 1,000, between the periods 2000 and 2019, at the level of Brazil and the Northeast Macroregion.	- Mortality information system. http://tabnet.datasus.gov.br/cgi/deftohtm.exe?sim/cnv/obt10uf.def - Live birth information system. http://tabnet.datasus.gov.br/cgi/deftohtm.exe?sinasc/cnv/nvuf.def
Childhood mortality coefficient	Dependent	Number of deaths in children under 5 years of age in a given locality divided by the total number of live births in a given locality, multiplied by 1,000 between the periods 2000 and 2019, at the level of Brazil and the Northeast Macroregion.	- Mortality information system. http://tabnet.datasus.gov.br/cgi/deftohtm.exe?sim/cnv/obt10uf.def - Live Birth information system. http://tabnet.datasus.gov.br/cgi/deftohtm.exe?sinasc/cnv/nvuf.def
Mortality coefficient (5–14 years)	Dependent	Number of deaths between the ages of 5 and 14 in a given locality divided by the number of residents in that locality, multiplied by 100,000, between 2000 and 2019, for Brazil and the Northeast Macroregion.	SIM—DATASUS; population estimates—IBGE
Race/color	Independent	Classification according to IBGE categories (white, black, brown, yellow, indigenous. ignored)	- Mortality Information System. http://tabnet.datasus.gov.br/cgi/deftohtm.exe?sim/cnv/obt10uf.def - Resident Population. http://tabnet.datasus.gov.br/cgi/deftohtm.exe?ibge/cnv/popsvsbr.def
Sex	Independent	Male, female, unknown	- Mortality Information System. http://tabnet.datasus.gov.br/cgi/deftohtm.exe?sim/cnv/obt10uf.def
Age group	Independent	Under 1 year old; under 5 years old; 5–14 years old	- Mortality Information System. http://tabnet.datasus.gov.br/cgi/deftohtm.exe?sim/cnv/obt10uf.def
Underlying cause of death (ICD-10)	Independent	Classification of underlying causes of death according to ICD-10 chapters	- Mortality Information System. http://tabnet.datasus.gov.br/cgi/deftohtm.exe?sim/cnv/obt10uf.def

The descriptive analysis of the data followed standards established for quantitative time series research. Initially, the data was extracted from DATASUS and then exported to *Microsoft Excel 2013*. At this stage, absolute and relative values were calculated, and graphs were drawn up to represent the data situation.

To analyze the statistical trend, we used the Joinpoint Regression Program software, version 5.0.2, which applies the linear regression coefficient estimate to identify statistically significant inflection points in the time series (17).

The information was entered into Excel by annual rate and categorized according to the type of variable and age group analyzed. The data was processed as follows:

(a). Separation of data by age group, year, race/color, number of deaths, underlying cause of mortality, and type of variable, for both Brazil and the Northeast Macroregion.
(b). Systematic recording of each stage of data collection, categorization of variables, and transposition of information into structured spreadsheets.
(c). Calculation of the mortality coefficients: infant (under 1 year old), childhood (under 5 years old), and children aged 5–14, as well as the generation of graphs and the calculation of percentages according to the variable's race/color, sex, and underlying cause of death—ICD-10.

Concerning time trend analysis, Joinpoint identifies changes in the slopes of the curves over time utilizing joinpoints using the Monte Carlo permutation method to verify the statistical significance of the changes observed. The model also allows you to choose between different error distributions, such as Poisson, when applicable (18). In the context of this analysis, two main indicators are used: APC (Annual Percentage Change) which estimates the annual percentage change in rates over time, indicating specific changes year on year. It is calculated using a log-linear regression model, in which the rate for one year is multiplied by a constant factor to obtain the rate for the next year. The AAPC (Average Annual Percentage Change), on the other hand, represents a weighted average of each APC within the specified time interval, providing an overview of the trend over the evaluated period (17).

This study did not require approval from the Research Ethics Committee (CEP), since it used data in the public domain, as provided for in Law No. 14.874 of 2024, which establishes research with human beings and implements the National Ethics System, in addition to the Access to Information Law No. 12.527 of 2011 (19, 20).

## 3 Results

The analysis of the 20 years investigated reveals that, in Brazil, there were a total of 1,259,615 deaths in children up to 14 years of age. Of these, 428,041 lived in the Northeast Macroregion. From 2000 to 2019, there was a significant change in the mortality coefficients for infants (under 1 year old), children (under 5 years old) and children aged 5–14.

As for infant mortality, the rates were consistently higher than those of child mortality, both in Brazil and in the Northeast Macroregion, as was expected, considering that the former includes the latter. In Brazil, the IMR fell from 26.10 to 12.40 deaths per thousand LB, while the IMRf fell from 30.10 to 14.10 deaths per thousand LB. In the Northeast Macroregion, the IMR fell from 35.90 to 13.70 deaths per 1,000 LB and the IMRf from 41.20 to 15.80 deaths per 1,000 LB.

It was also possible to identify a reduction in mortality rates for children aged 5–14. However, these rates remained higher at the end of the historical series concerning the IMR and IMRf, reaching rates of 25.01 and 28.10 deaths per 100,000 inhabitants aged 5–14 in Brazil and 28.10 deaths per 100,000 inhabitants in the same age group in the Northeast in 2019.

Over the entire period analyzed, the Northeast maintained rates higher than the national average, reinforcing the existence of significant regional disparities in child health indicators. This pattern is compatible with the context of social, economic, and access to health services inequalities between Brazilian macro-regions, as illustrated in Table 2.

**Table 2:** Distribution of mortality rates in Brazil and the northeast macro-region, 2000 to 2019.

Year	IMR (<1 year) Brazil	IMR (<1 year)—Northeast	TMIf (<5 years) Brazil	TMInf (<5 years)—Northeast	TM 5–14 years—Brazil	MT 5–14 years—Northeast
2000	26.10	35.90	30.10	41.20	33.35	33.34
2001	24.90	33.40	28.70	38.30	32.76	32.38
2002	24.90	33.40	27.20	35.50	33.01	33.28
2003	23.40	30.80	26.10	33.80	32.14	32.08
2004	21.50	27.80	25.00	32.10	31.46	32.21
2005	20.40	25.90	23.70	29.90	30.72	30.96
2006	19.60	24.80	22.70	28.50	31.21	31.31
2007	18.60	23.20	21.70	26.80	30.27	31.70
2008	17.70	21.80	20.60	25.20	30.76	34.04
2009	16.80	20.30	19.60	23.50	30.32	31.85
2010	16.00	19.10	18.60	22.10	29.71	32.27
2011	15.30	18.00	17.70	20.70	29.42	31.03
2012	13.50	15.00	15.60	17.40	29.94	31.76
2013	13.40	15.50	15.60	17.80	28.86	30.89
2014	12.90	14.50	14.90	16.70	28.73	31.16
2015	12.40	14.00	14.30	16.00	26.61	28.60
2016	12.70	14.50	14.90	16.80	27.03	29.99
2017	12.40	14.10	14.40	16.30	26.25	29.48
2018	12.20	13.50	14.20	15.70	25.02	28.12
2019	12.40	13.70	14.40	15.80	25.01	28.10

**Figure 1:**
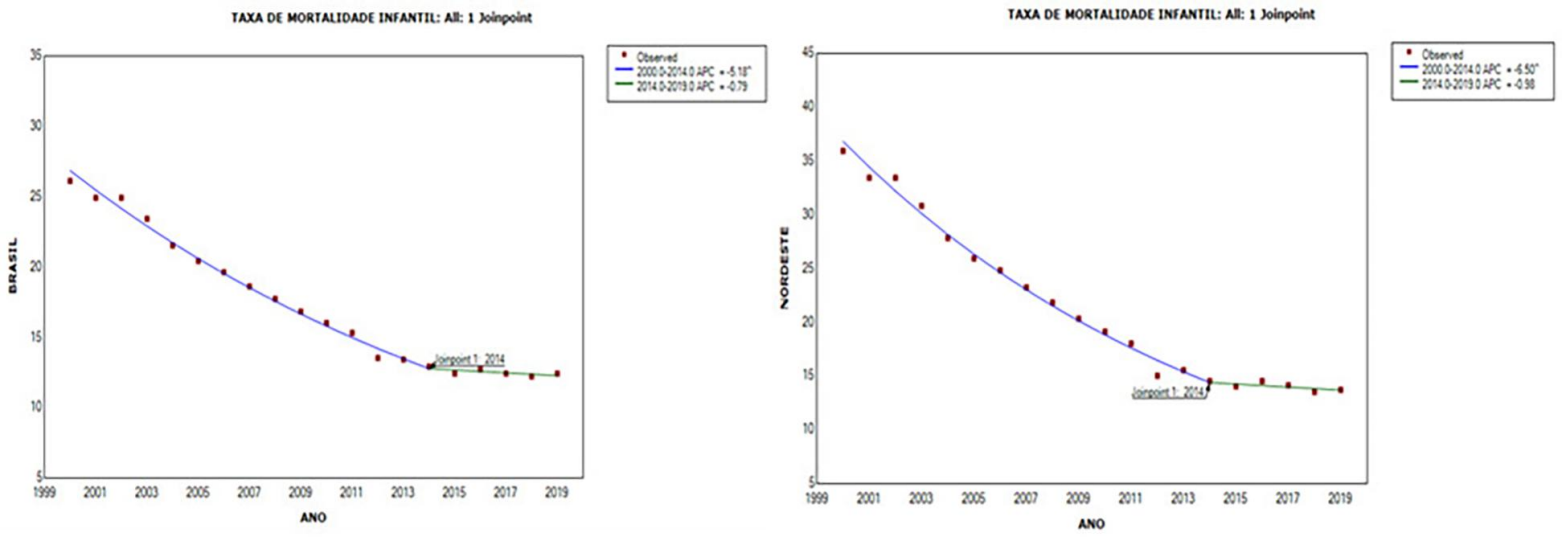
Trend in the infant mortality rate (under 1 year) in Brazil and the northeast region, 2000 to 2019.

Figure 1 shows a statistically significant downward trend in the IMR in Brazil and the Northeast, respectively, *APC* = *−5.2 (95%CI: −5.5; −4.9*) and *APC* = *−6.50 (95%CI: −6.9 and −6.1), in* the period between 2000 and 2014, with a *joinpoint* in 2014, when the mortality coefficients began to show stability, with no statistically significant variations until 2019.

**Figure 2:**
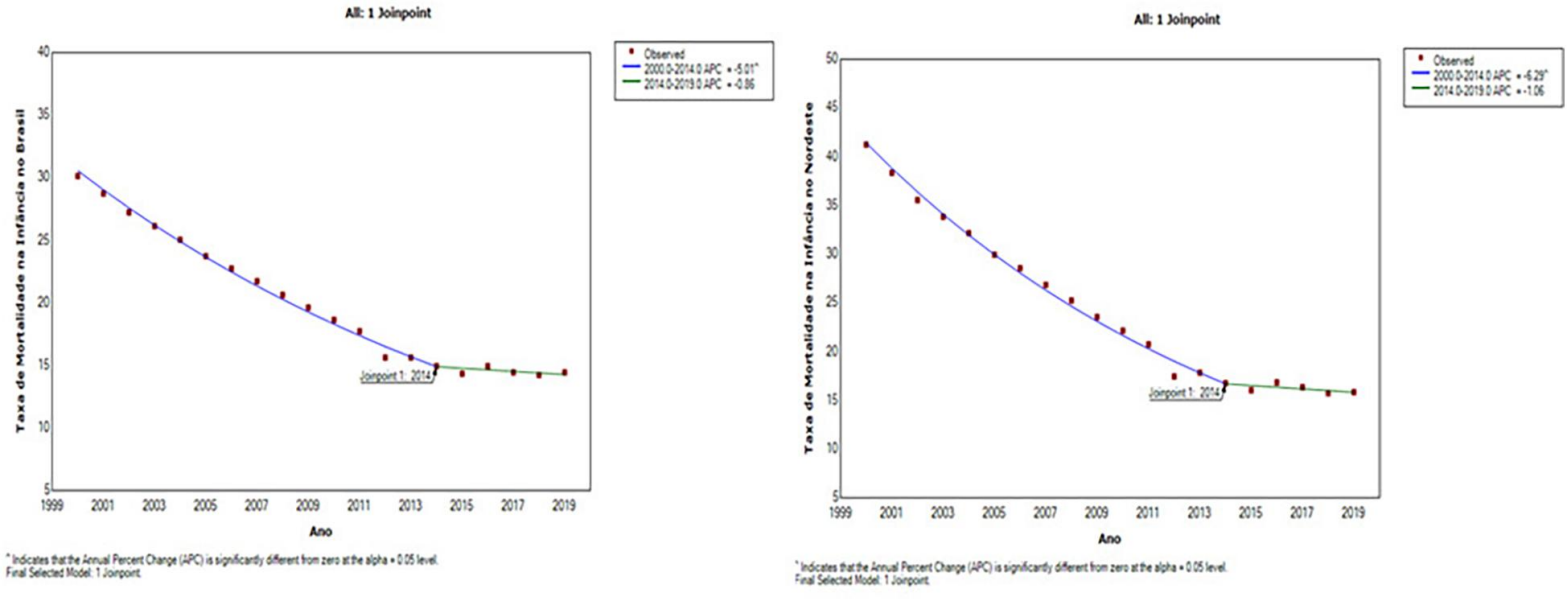
Trend in the under-five mortality rate in Brazil and the northeast macro-region, 2000 to 2019.

Figure 2 also shows a statistically significant reduction in the under-five mortality rate over the same time period (2000–2014). In Brazil, the downward trend showed an *APC* = *−5.01 (95%CI: −5.3 and −4.7),* while in the Northeast Macroregion, the reduction was even more pronounced, with an *APC* = *−6.29 (95%CI: −6.7 and −5.9)*. In the subsequent period (2014–2019), although mortality rates continued to fall, the variations were not statistically significant, signaling a period of stagnation in the reduction of mortality among children under 5 years of age.

**Figure 3:**
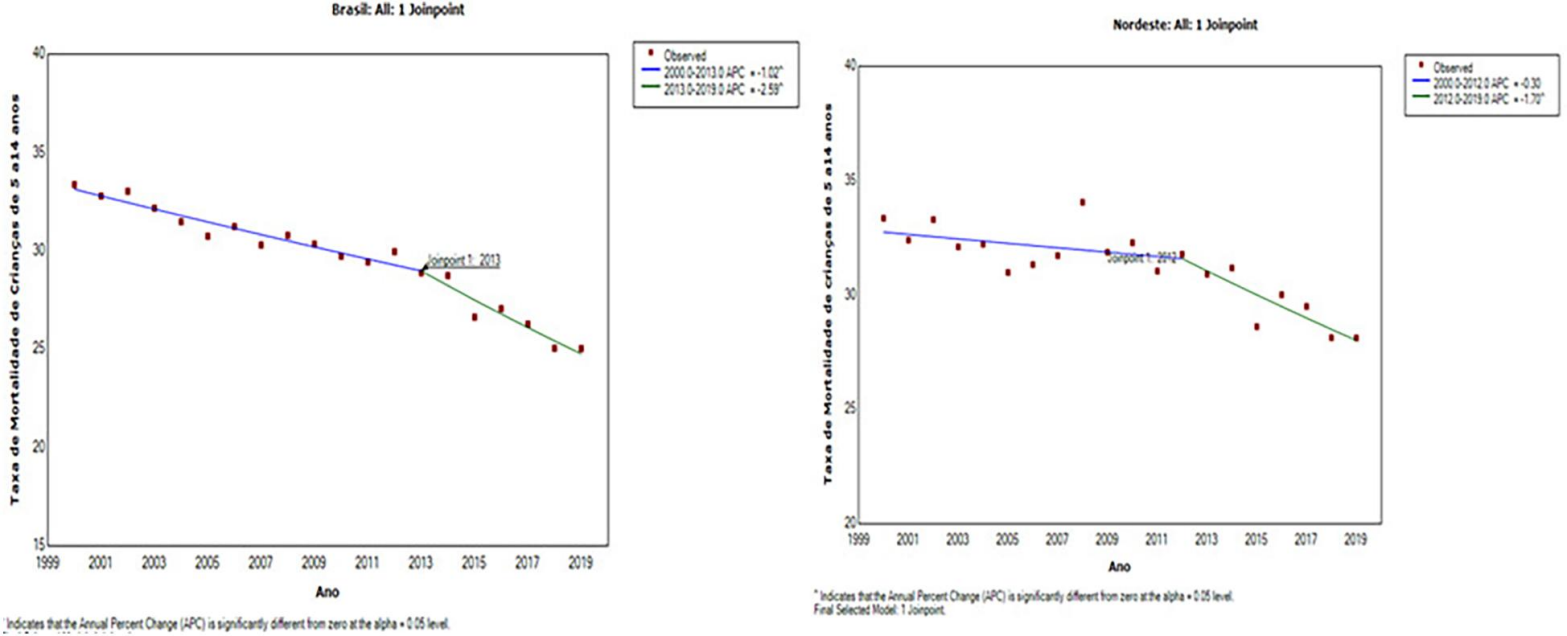
Trend in the mortality rate for children (5 to 14 years) in Brazil and the Northeast Macroregion, 2000 to 2019.

Although the mortality rate for children aged 5–14 showed a downward trend throughout the study period, it showed a less marked decline than the IMR and IMRf. In Brazil, this reduction was statistically significant in both periods: between 2000 and 2013, *APC* = *−1.02 (CI:−1.3;−0.8)* and between 2013 and 2019, *APC* = *−2.6 (CI:−3.4;−1.8).* In the Northeast, the downward trend was only confirmed in the most recent period, between 2012 and 2019 with *(APC* = *−1.7; 95% CI −2.8 and −0.6)*, as shown in Figure 3.

Concerning the analysis of the race/color variable, national data between 2000 and 2019 reveal a predominance in the number of deaths in children under 1 year old among white children, 369,584 (40.61%), followed by brown children, 353,624 (38.86%). This pattern was maintained among children under the age of 5. However, when considering the black population group (brown and black), the percentage was higher: 41.38% (*N* = 376,566) in children under 1 year old and 41.95% (*N* = 446,448) in children under 5, according to Table 3.

**Table 3:** Distribution of child deaths by race/color and age group in Brazil and the Northeast Macroregion, 2000 to 2019.

Race/color	Deaths Brazil						Deaths northeast					
	<1 year		<5 years		5–14 years		<1 year		<5 years		5–14 years	
	*N*	%	*N*	%	*N*	%	*N*	%	*N*	%	*N*	%
White	369.584	40.61	433.239	40.71	77.483	39.66	58.368	18.63	70.442	19.3	12.480	19.77
Black	22.942	2.52	29.214	2.75	10.980	5.62	6.915	2.21	9.195	2.52	3.518	5.57
Yellow	2.176	0.24	2.588	0.24	502	0.26	717	0.23	871	0.24	152	0.24
Brown	353.624	38.86	417.234	39.2	88.017	45.05	161.763	51.64	189.648	51.97	37.874	59.99
Indigenous	11.385	1.25	15.751	1.48	2.095	1.07	1.414	0.45	1.787	0.49	218	0.35
Unknown (a)	150.396	16.53	166.214	15.62	16.298	8.34	84.055	26.83	92.965	25.48	8.891	14.08
Total	910.107	100	1.064.240	100	195.375	100	313.232	100	364.908	100	63.133	100
Male	506.405	55.64	590.266	55.46	116.906	59.84	174.829	55.81	202.717	55.55	38.038	60.25
Female	398.254	43.76	468.482	44.02	78.437	40.15	135.005	43.10	158.769	43.51	25.077	39.72
Ign	5.448	0.60	5.492	0.52	32	0.02	3.398	1.08	3.422	0.94	18	0.03
Total	910.107	100	1.064.240	100	195.375	100	313.232	100	364.908	100	63.133	100

In the Northeast Macroregion, the profile of infant mortality is particular. The highest number of deaths was among brown children, 161,763 (51.67%), followed by white children, 58,368 (18.63%). Similarly, mortality among children under 5 was higher among brown children, 189,648 (51.97%).

In children aged 5–14, although the absolute number was lower in both Brazil and the Northeast, there was a change in the racial profile of deaths. In Brazil, brown children accounted for the majority of deaths in this age group, 37,874 (59.99%), followed by white children, 12,480 (19.77%). When the deaths of brown and black children are added together, the percentage of fatalities reaches 50.67% (*N* = 98,997) in Brazil and 65.56% (*N* = 41,392) in the Northeast.

Stratification by sex also reveals inequalities. In all age groups in the localities, the number of deaths was higher in the male population. In Brazil, over the 20 years investigated, the highest concentration of deaths occurred in children under 5, representing a total of 1,058,748, of which 590,266 (55.75%) were male and 468,482 (44.25%) female. In the Northeast Macroregion, this pattern was repeated, with 361,486 deaths in children under 5, of which 202,717 (56.08%) occurred in males and 158,769 (43.92%) in females, representing approximately one-third of child deaths in Brazil, as shown in Table 3.

The analysis of child deaths, according to ICD-10 chapters, in Brazil and the Northeast Macroregion, between 2000 and 2019, shows that conditions originating in the perinatal period (Chapter VI) were the leading cause of mortality in children. In Brazil, this condition was responsible for 523,826 deaths, almost all of which occurred in children under the age of 1 (*N* = 522,602; 57.42%). In the Northeast Macroregion, this pattern continued, with 181,296 deaths, also concentrated in children under 1 year old. This cause alone accounts for almost half of child deaths in both locations.

In second place were congenital malformations, deformities, and chromosomal anomalies (Chapter XVII), both in Brazil (*N* = 182,688) and in the Northeast Macroregion (*N* = 50,601), primarily affecting children under 1 year of age. In Brazil, this category accounted for 17.58% of deaths in children under 1 and 3.85% in the 5–14 age group. In the Northeast, the proportion was 14.12% and 3.43%, respectively.

External causes of morbidity and mortality (Chapter XX) appear as the third leading cause of death in children aged 0–14. In Brazil, 129,724 deaths were recorded, of which (*N* = 76,961; 39.39%) occurred among children aged 5–14. In the Northeast, this proportion was even higher in this age group, with 24,040 deaths (38.08%) out of a total of 37,523. This data shows that external causes are more relevant at older ages.

Concerning diseases of the respiratory system (Chapter X), they ranked fourth in terms of causes of death in Brazil (91,115), especially among children under 1 (*N* = 51,513; 5.66%) and under 5 (*N* = 77,224; 7.26%). In the Northeast, however, syndromes, signs, and abnormal clinical and laboratory findings (Chapter XVIII) were more relevant, with 35,733 deaths, 23,370 of which (7.46%) were in children under 1 year old.

In fifth place were some infectious and parasitic diseases (Chapter I), both in Brazil (*N* = 84,973) and in the Northeast (*N* = 34,981), mainly affecting children under 5. Also worth mentioning are diseases of the nervous system (Chapter VI) and neoplasms (Chapter II), which, although less representative of total deaths, still show significant numbers. 42,376 and 40,964 deaths were recorded in Brazil, respectively, and 12,482 and 12,180 in the Northeast, respectively.

Tables 4, 5 show the main causes of mortality.

**Table 4:** Distribution of child deaths by ICD-10 chapter and age group in Brazil, 2000–2019.

ICD-10 chapter	<1 year		<5 years		5–14 years		Total *N*	
	*N*	%	*N*	%	*N*	%	*N*	%
XVI. Some conditions originating in the perinatal period	522.602	57.42	523.511	49.19	315	0.16	523.826	41.58
XVII. Malf cong deformid and chromosomal anomalies	159.990	17.58	175.171	16.46	7.517	3.85	182.688	14.50
XX. External causes of morbidity and mortality	21.146	2.32	52.763	4.96	76.961	39.39	129.724	10.30
X. Diseases of the respiratory system	51.513	5.66	77.224	7.26	13.891	7.11	91.115	7.23
I. Some infectious and parasitic diseases	53.010	5.83	72.520	6.81	12.453	6.37	84.973	6.74
XVIII. Symptoms. signs and abnormal clinical and laboratory findings	48.820	5.36	64.374	6.05	13.365	6.84	77.739	6.17
VI. Diseases of the nervous system	11.862	1.30	24.847	2.34	17.529	8.97	42.376	3.36
II. Neoplasms (tumors)	3.026	0.33	15.076	1.42	25.888	13.25	40.964	3.25
IV. Endocrine. nutritional and metabolic diseases	14.901	1.64	21.168	1.99	4.253	2.18	25.421	2.02
IX. Diseases of the circulatory system	8.220	0.90	13.249	1.25	9.385	4.80	22.634	1.80
XI. Diseases of the digestive system	6.719	0.74	9.922	0.93	4.408	2.26	14.330	1.14
III. Diseases of the blood. blood organs and immune disorders	4.701	0.52	8.280	0.78	3.881	1.99	12.161	0.97
XIV. Diseases of the genitourinary system	2.459	0.27	4.091	0.38	2.434	1.25	6.525	0.52
XIII. Diseases of the musculoskeletal system and connective tissue	210	0.02	1.018	0.10	1.587	0.81	2.062	0.16
XII. Diseases of the skin and subcutaneous tissue	631	0.07	475	0.05	573	0.29	1.591	0.13
VIII. Diseases of the ear and mastoid apophysis	231	0.03	368	0.04	216	0.11	584	0.05
V. Mental and behavioral disorders	27	0.003	128	0.01	372	0.19	500	0.04
XV. Pregnancy. childbirth and puerperium	–	–	–	–	319	0.16	319	0.03
VII. Diseases of the eye and appendages	39	0.004	55	0.01	28	0.01	83	0.01
Total	910.107	100.00	1.064.240	100.00	195.375	100.00	1.259.615	100.00

**Table 5:** Distribution of child deaths by ICD-10 chapter and age group in the northeast macroregion, 2000–2019.

ICD-10 chapter	<1 year		<5 years		5–14 years		Total *N*	
	*N*	%	*N*	%	*N*	%	*N*	%
XVI. Some conditions originating in the perinatal period	180.951	57.77	181.238	49.67	58	0.09	181.296	42.35
XVII.Malf cong deformid and chromosomal anomalies	44.238	14.12	48.434	13.27	2.167	3.43	50.601	11.82
XX. External causes of morbidity and mortality	4.099	1.31	13.483	3.69	24.040	38.08	37.523	8.77
XVIII.Sint signs and abnormal ex-clinical and laboratory findings	23.370	7.46	30.175	8.27	5.558	8.8	35.733	8.35
I. Some infectious and parasitic diseases	23.752	7.58	30.859	8.46	4.122	6.53	34.981	8.17
X. Diseases of the respiratory system	17.035	5.43	25.897	7.1	4.571	7.24	30.468	7.12
VI. Diseases of the nervous system	3.520	1.12	7.353	2.02	5.129	8.12	12.482	2.92
II. Neoplasms (tumors)	977	5.74	4.595	1.26	7.585	12.01	12.180	2.85
IV. Nutritional and metabolic endocrine diseases	6.961	2.22	9.276	2.54	1.484	2.35	10.760	2.51
IX. Diseases of the circulatory system	2.542	0.81	4.241	1.16	3.475	5.5	7.716	1.80
III. Blood diseases. blood organs and immune disorders	2.229	0.71	3.732	1.02	1.402	2.22	5.134	1.20
XI. Diseases of the digestive system	2.304	0.74	3.419	0.94	1.486	2.35	4.905	1.15
XIV. Diseases of the genitourinary system	848	0.27	1.484	0.41	1.011	1.6	2.495	0.58
XIII Musculoskeletal system and connective tissue diseases	68	0.02	145	0.04	479	0.76	624	0.15
XII. Diseases of the skin and subcutaneous tissue	230	0.07	376	0.1	215	0.34	591	0.14
VIII Diseases of the ear and mastoid apophysis	83	0.03	125	0.03	72	0.11	197	0.05
V. Mental and behavioral disorders	11	0.004	53	0.01	137	0.22	190	0.04
XV. Pregnancy. childbirth and puerperium	-	-	-	-	132	0.21	132	0.03
VII. Diseases of the eye and appendages	14	0.004	23	0.01	10	0.02	33	**0.01**
Total	313.232	100.00	364.908	100.00	63.133	100.00	428.041	100.00

## 4 Discussion

Our study revealed that Brazil and the Northeast Macroregion showed a downward mortality trend among all age groups during the twenty years analyzed. During this period, the Infant Mortality Rate (IMR) and the Child Mortality Rate (IMRf) fell significantly in both territories. Falls were also observed in the mortality rates of children aged 5–14, indicating a positive impact of public health policies over the years. These data highlight the importance of robust health policies and strategies, particularly in historically vulnerable regions such as the Northeast, and reveal significant territorial disparities.

The historical trajectory of the reduction in mortality is confirmed by strategic and assertive public policies such as the expansion of access to Primary Care, the creation of the National Immunization Program (PNI) in 1973, the Prenatal and Birth Humanization Program (PHPN), the Bolsa Família Program (PBF) in 2003, the Pact for the Reduction of Maternal and Neonatal Mortality in 2004, the Rede Cegonha Program in 2011, the National Policy for Comprehensive Child Health Care (PNAISC) in 2015, among others (21). However, even with the notorious advances, the lower infant mortality rates in Brazil are still far from those observed in countries with a high Human Development Index (HDI), which register around three deaths per thousand live births (5).

When the Annual Percentage Change (APC) is analyzed, it becomes apparent that the IMR and IMNF rates exhibit similar behavior, with a greater percentage reduction from 2000 to 2014, followed by a stationary trend between 2014 and 2019. This deceleration indicates that although public policies have been effective, after the inflection point, they have not been sufficient to maintain the progressive pace of these changes.

This stationary behavior of mortality may be related to economic crises, which have a more severe impact on low- and middle-income countries, resulting in damage to child health (22, 23). Since 2014, Brazil has faced one of the most severe economic crises in its recent history, marked by a decline of more than 8% in Gross Domestic Product (GDP), rising unemployment, and increased poverty (23–25). The political crisis culminated in the impeachment of the elected president, and, in 2016, the controversial Constitutional Amendment Number 95 was approved, implemented in 2017, which imposed a ceiling on federal spending for 20 years, compromising resources for health and social security (26, 27). As a result, social programs such as the Bolsa Família Program (PBF) and the Family Health Strategy (ESF), which are fundamental in reducing child mortality, were undermined and, as a consequence, there was an increase in poverty due to the strengthening of the fiscal austerity measures adopted (28). According to Aransiola et al. (29), if income transfer, social security, and Primary Health Care policies had been expanded, it would have been possible to avoid up to 182,531 deaths of children under 5 between 2000 and 2030, compared to a scenario of fiscal austerity.

Although substantial progress has been made in the fight against child mortality in Brazil and the Northeast, the slowdown observed since 2014 highlights the importance of maintaining and continuously strengthening public health policies to ensure the prevention of avoidable deaths. Issues such as the change in socio-economic profile, access to contraceptive methods and changes in family planning policies also influence the number of births, which fell from 3.6 million in 2000 to 2.6 million in 2022. The persistence of territorial inequalities means that many municipalities have not yet reached target 4 set in the MDGs (30, 31).

For the mortality rate among children aged 5–14, there was statistical significance both before and after the inflection point. In the Northeast, there was a greater reduction in the APC after this point. The slower reduction in the rate may be due to the lower risk of preventable causes of death typical of the childhood period. This reinforces the need to broaden approaches to this group, including traditional health determinants.

According to Fadel et al. (8), in their study, the majority of deaths recorded between 5 and 14 years of age in the country were caused by preventable or treatable conditions, which points to the need for specific public policies aimed at children and adolescents. This highlights the need for targeted public policies focused on children and adolescents to strengthen the fight against inequalities.

Another point worth highlighting is that health services still require expansion and strengthening. Factors linked to child mortality affect their health conditions and are directly related to the quality of Family Health Strategy (FHS) services (32). Despite the importance of Primary Care, around 34% of the population is still not covered by the FHS, which corresponds to 72.69 million people, with the North and Northeast being the regions with the highest percentage of socially vulnerable municipalities, as well as having low coverage rates (11).

Risk stratification is essential in childcare (33). Regarding the incompleteness of the data, a study by researchers Melo and Valongueiro (34) suggests that proper recording is crucial for understanding events and planning effective intervention and prevention strategies. However, there are still significant flaws on the part of some professionals who persist in attributing little relevance to some topics in the death certificate, which motivated the Ministry of Health to make it mandatory to fill in the race/color attribute in the SUS information systems (35). However, even with the current ordinance, little progress has been made, which highlights the persistence of institutional racism (36, 37).

Analysis of mortality data indicates disparities in terms of racial and regional characteristics, and by cause of death. The country also experiences high inequality, generated by class, gender, and race or ethnicity (38). Compared to children of white mothers, for example, the mortality risk rate for children aged 1–4 was 65% higher among children of black mothers and 43% higher among children of brown mothers. The raw data also shows that children of indigenous mothers have a 31-, 11-, and 52-times higher proportion of deaths from diarrhea, malnutrition, and flu/pneumonia than children of white mothers, respectively. In the case of children of black mothers, the risk of death in children up to 5 years old was approximately twice as high for diarrhea, influenza/pneumonia, and around three times as high for malnutrition compared to children of white mothers (39).

It is also noteworthy that the drop in the proportion of deaths among indigenous children does not follow the same speed of reduction when compared to the resident population (40, 41). According to the IBGE (30), the highest percentage of indigenous people is between the ages of 0 and 14 (29.95%).

As for mortality by sex, there is a predominance of male deaths in all age groups in the country and macro-region, possibly due to greater biological vulnerability to diseases linked to external causes, diarrhea, hemorrhages, and pneumonia (38).

The research conducted by Moreira, Oliveira, and Andrade (42) suggests that perinatal causes are a significant factor contributing to infant mortality in the Northeast Macroregion. In this analysis, hospital morbidity and mortality data showed that perinatal diseases and respiratory disorders were the most common conditions, especially bacterial septicemia among infants. In the study by Alves, Fontenelle and Sarti (43), external causes, such as traffic accidents and homicides, are the main mortality factors among children aged 5–14 in Brazil. In the study by Fadel et al. (8), the majority of deaths between 2005 and 2016 among children aged 5–14 in India, China, Brazil and Mexico were due to preventable or treatable conditions. Therefore, it is necessary to expand some specific global targets for this audience.

This study has limitations. Firstly, using a regional scale can mask disparities at more aggregate levels, and it also restricts some comparisons at the country and macro-regional levels. However, the analysis effectively captures relevant variations to be considered in the context of mortality. Secondly, as this is a study using secondary data, some of the projected information may be incomplete or require updating. However, it is the most reliable information available for population analysis.

Few studies address the mortality of children aged 5–14, which indicates the need for more research in this area. The findings of this study, in line with other literature, indicate significant progress in reducing the mortality rate of children in different age groups; however, a slowdown in this annual percentage rate began in mid-2014. These processes may indicate influences on effective health policies and strategies, but they also point to recent setbacks marked by austerity policies. Territorial inequalities persist and continue to negatively influence the living conditions of children in Brazil and the Northeast.

## 5 Conclusion

This study presents key findings on child mortality rates in Brazil and the Northeast Macroregion from 2000 to 2019. Significant decreases over the last two decades can be attributed to effective public policies, including the expansion of Primary Care, income transfer programs, and the National Immunization Program. Despite this, socio-economic and regional inequalities, especially in the Northeast Macroregion, persist as obstacles to sustainable progress, directly impacting child mortality.

Analyzing mortality as a reflection of social, economic, and political conditions necessitates an understanding that child health is deeply intertwined with the fulfillment of fundamental rights. The literature and data indicate that austerity cycles and the reduction of redistributive policies have a more profound impact on vulnerable populations, exacerbating racial, class, and spatial inequalities. This manifests itself in various patterns of mortality that cannot be explained by biomedical or individual variables alone.

The economic and political crisis experienced by Brazil since 2014 has slowed down progress on child mortality. It has also weakened the Unified Health System (SUS), compromising the sustainability of essential policies such as the Family Health Strategy and the Bolsa Família Program. Another limitation of this study is the lack of more disaggregated analysis by municipality, specific age groups, and markers such as race/color, maternal schooling, and access to essential services, which could deepen the understanding of health inequities. Furthermore, the exclusive use of quantitative data limits the knowledge of the subjective and contextual dimensions of the experience of these preventable deaths.

This reaffirms the need for intersectoral public policies, with an emphasis on the social determinants of health, territorial equity, and the strengthening of the SUS and social protection. Ensuring the right to life of children and adolescents requires a political commitment and sustained investment in health, education, sanitation, and food security, especially in contexts of historical inequality, such as the Brazilian Northeast.

It also reinforces the importance of academia, government and civil society working together to mitigate inequalities, consolidate surveillance systems and make progress on the quality of data and interventions. Ultimately, through strategic and coordinated planning that incorporates intersectorality, equity, and efficiency in the management of public resources, it will be possible to enhance health indicators.

## Data Availability

The original contributions presented in the study are included in the article/Supplementary Material, further inquiries can be directed to the corresponding author.

## References

[1] UN. UN: Brazil Meets Infant Mortality Reduction Target. New York: Civil House (Presidency of the Republic, Brazil) (2015). Available online at: https://www.gov.br/casacivil/pt-br/assuntos/noticias/2015/setembro/onu-brasil-cumpre-meta-de-reducao-da-mortalidade-infantil (Accessed December 22, 2024).

[2] SVS. Infant Mortality in Brazil. Brasília, DF: Ministry of Health (2021). Available online at: https://www.gov.br/saude/pt-br/centrais-de-conteudo/publicacoes/boletins/epidemiologicos/edicoes/2021/boletim_epidemiologico_svs_37_v2.pdf (Accessed October 24, 2024).

[3] Abrinq F. Scenario of Childhood and Adolescence in Brazil. São Paulo, SP: Abrinq Foundation (2023). Available online at: https://observatoriocrianca.org.br/system/library_items/files/000/000/035/original/cenario-da-infancia-e-adolescencia-no-brasil-2023.pdf.pdf?1678125969 (Accessed January 1, 2024).

[4] Abrinq F. Scenario of Childhood and Adolescence in Brazil. São Paulo, SP: Abrinq Foundation (2024). Available online at: https://fadc.org.br/sites/default/files/2024-03/fundacao-abrinq-cenario-2024.pdf (Accessed January 1, 2024).

[5] United Nations Development Programme. Human Development Report 2014: Sustaining Human Progress: Reducing Vulnerabilities and Building Resilience. New York: United Nations Development Programme (2014). Available online at: https://hdr.undp.org/content/human-development-report-2014 (Accessed January 1, 2024).

[6] UN—IGME. Levels & Trends in Child Mortality Report 2023. New York: United Nations Children’s Fund (UNICEF) (2023). Available online at: https://brasil.un.org/sites/default/files/2024-03/UNIGME-2023-Child-Mortality-Report.pdf (Accessed January 1, 2024).

[7] UN—IGME. Levels & Trends in Child Mortality. New York: United Nations Children’s Fund (2017). Available online at: https://www.unicef.org/media/48871/file/Child_Mortality_Report_2017.pdf (Accessed November 10, 2024).

[8] Fadel SA Boschi-Pinto C Yu S Reynales-Shigematsu LM Menon GR Newcombe L Trends in cause-specific mortality among children aged 5–14 years from 2005 to 2016 in India, China, Brazil, and Mexico: an analysis of nationally representative mortality studies. Lancet. (2019) 1:1119–27. 10.1016/S0140-6736(19)30220-X PMC6418656 30876707

[9] Victora CG Hartwig FP Vidaletti LP Martorell R Osmond C Richter LM Effects of early-life poverty on health and human capital in children and adolescents: analyses of national surveys and birth cohort studies in LMICs. Lancet. (2022) 399(10336):1741–52. 10.1016/S0140-6736(21)02716-1 35489358 PMC9061872

[10] UN. Situation of the World’s Poorest Children has Seen Little Progress in the Last 30 Years. New York: United Nations (UN) (2019). Available online at: https://news.un.org/pt/story/2019/11/1694741 (Accessed December 24, 2024).

[11] Institute for Health Policy Studies. Annual Report 2022: Health in Economic and Social Order [Internet]. São Paulo: IEPS (2022). p. 80. Available online at: https://ieps.org.br/wp-content/uploads/2023/03/IEPS_Annual_Report_2022.pdf (Accessed January 1, 2024).

[12] Instituto Trata Brasil. States in the North and Northeast are among the Five with the Greatest Deprivation of Sewage Collection. São Paulo, SP: Trata Brasil Institute (2024). Available online at: https://tratabrasil.org.br/estados-do-norte-e-do-nordeste-estao-entre-os-cinco-com-as-maiores-privacoes-de-coleta-de-esgoto/ (Accessed December 29, 2024).

[13] Dias BAS Santos-Neto ET Andrade MAC Zandonade E. Spatial analysis of preventable infant deaths in Espírito Santo, Brazil, 2006–2013. EHSJ. (2019) 28(3):1–11. 10.5123/S1679-49742019000300001 31508713

[14] Wiener N. Extrapolation, Interpolation, and Smoothing of Stationary Time Series. Cambridge, MA: MIT Press (1966). Available online at: https://mitpress.mit.edu/9780262730051/extrapolation-interpolation-and-smoothing-of-stationary-time-series/ (Accessed May 16, 2024)

[15] Antunes JLF Cardoso MRA. Use of time series analysis in epidemiological studies. Epidemiol Health Serv. (2015) 24:565–76. 10.5123/S1679-49742015000300024

[16] IBGE. In 2022, the Number of Births Falls for the Fourth Year and Reaches the Lowest Level Since 1977. Rio de Janeiro, RJ: Brazilian Institute of Geography and Statistics (IBGE) (2025). Available online at: https://agenciadenoticias.ibge.gov.br/agencia-noticias/2012-agencia-de-noticias/noticias/39560-em-2022-numero-de-nascimentos-cai-pelo-quarto-ano-e-chega-ao-menor-patamar-desde-1977 (Accessed February 17, 2025).

[17] NIH. Joinpoint Regression Program. United States of America: National Cancer Institute (2024). Available online at: https://surveillance.cancer.gov/joinpoint/ (Accessed January 1, 2024).

[18] Kim HJ Fay MP Feuer EJ Midthune DN. Permutation tests for joinpoint regression with applications to cancer rates. Stat Med. (2000) 19(3):335–51. 10.1002/(SICI)1097-0258(20000215)19:3<335::AID-SIM336>3.0.CO;2-Z 10649300

[19] Brazil. Presidency of the Republic. Law No. 14.874, of May 28, 2024. Provides for the National Policy to Support People with Rare Diseases and amends Law No. 8,080, of September 19, 1990, to provide for the organization of the national network for the care of people with rare diseases within the Unified Health System (SUS). Diário Oficial da União [Internet], of May 29, 2024. Available online at: https://www.in.gov.br/en/web/dou/-/lei-n-14.874-de-28-de-maio-de-2024-

[20] Brazil. Presidency of the Republic. Law No. 12.527, of November 18, 2011. Regulates the constitutional right of access to public information and establishes the procedures to be followed by the Union, States, Federal District and Municipalities in order to ensure this right. Diário Oficial da União [Internet], of November 18, 2011. Available online at: http://www.planalto.gov.br/ccivil_03/_ato2011-2014/2011/lei/l12527.htm

[21] Brasil MDAS. National Policy for Comprehensive Child Health Care. Brasília, DF: Ministry of Health (2018). Available online at: https://www.gov.br/saude/pt-br/assuntos/saude-de-a-a-z/p/pnaisc (Accessed November 17, 2024).

[22] Oreiro JL. The great Brazilian recession: diagnosis and an economic policy agenda. Estudos Avançados. (2017) 31:75–88. 10.1590/s0103-40142017.31890009

[23] Skoufias E Shohei N Renata MG. Safeguarding Against a Reversal in Social Gains During the Economic Crisis in Brazil. Washington, DC: World Bank Group (2017).

[24] Rajmil L Fernández de Sanmamed MJ Choonara I Faresjö T Hjern A Kozyrskyj AL Impact of the 2008 economic and financial crisis on child health: a systematic review. Int J Environ Res Public Health. (2014) 11(6):6528–46. 10.3390/ijerph110606528 25019121 PMC4078594

[25] Gunnlaugsson G. Child health in Iceland before and after the economic collapse in 2008. Arch Dis Child. (2016) 101(5):489–96. 10.1136/archdischild-2014-307196 26471112

[26] Bitencourt LV Bitencourt CM. The austerity of constitutional amendment 95/2016 and the advance of the post-democratic state. Rev Direito Práxis. (2023) 14:139–64. 10.1590/2179-8966/2021/56212

[27] Vieira OV. A Batalha dos Poderes: Da Transição Democrática ao mal-estar constitucional. São Paulo, SP: Editora Schwarcz S.A (2018).

[28] Rasella D Basu S Hone T Paes-Sousa R Ocké-Reis CO Millett C. Child morbidity and mortality associated with alternative policy responses to the economic crisis in Brazil: a nationwide microsimulation study. PLoS Med. (2018) 15(5):e1002570. 10.1371/journal.pmed.1002570 29787574 PMC5963760

[29] Aransiola TJ Cavalcanti D Ordoñez JA Hessel P Moncayo AL Chivardi C Current and projected mortality and hospitalization rates associated with conditional cash transfer, social pension, and primary health care programs in Brazil, 2000–2030. JAMA Netw Open. (2024) 7(4):e247519. 10.1001/jamanetworkopen.2024.7519 38648059 PMC11036142

[30] IBGE. Panorama of the 2022 Census. Rio de Janeiro, RJ: Brazilian Institute of Geography and Statistics (IBGE) (2024). Available online at: https://censo2022.ibge.gov.br/panorama/ (Accessed December 30, 2024).

[31] World Bank Group. The World Bank in Brazil. Washington, DC: World Bank Group (2024). Available online at: https://www.worldbank.org/en/country/brazil (Accessed February 17, 2025).

[32] Pinto LF Giovanella L. From the program to the family health strategy: expanding access and reducing hospitalizations for primary care sensitive conditions (ICSAB). Ciência Saúde Coletiva. (2018) 23:1903–14. 10.1590/1413-81232018236.05592018 29972498

[33] Brasil MDAS. Death Surveillance. Brasília, DF: Ministry of Health (Brazil) (2021). Available online at: https://www.gov.br/saude/pt-br/composicao/svsa/verificacao-de-obitos/vigilancia-do-obito (Accessed November 17, 2024).

[34] Melo GBT Valongueiro S. Incompleteness of external cause death records in the mortality information system in pernambuco, Brazil, 2000–2002 and 2008–2010. Epidemiol Serv Saúde. (2015) 24(4):651–60. 10.5123/S1679-49742015000400007

[35] Brasil MDAS. Filling in Race/color Becomes Mandatory in SUS Information Systems. Brasília, DF: Ministry of Health (Brazil) (2017). Available online at: https://www.gov.br/saude/pt-br/assuntos/noticias/2017/fevereiro/preenchimento-da-raca-cor-se-torna-obrigatorio-nos-sistemas-de-informacao-do-sus (Accessed February 17, 2025).

[36] Werneck J. Institutional racism and the health of the black population. Saúde Sociedade. (2016) 25:535–49. 10.1590/s0104-129020162610

[37] Lopes F. Back to the beginnings: in defense of the SUS as an anti-racist policy. Boletim Análise Político Inst. (2021) 26:9–19. 10.38116/bapi26art1

[38] Alves LGR Guimarães RM. Race inequalities in maternal mortality in the city of Rio de Janeiro, Brazil: 2010–2019. J Br Med Assoc. (2021) 67(1):120–4. 10.1590/1806-9282.67.01.20200633 34161474

[39] Rebouças P Goes E Pescarini J Ramos D Ichihara MY Sena S Ethnoracial inequalities and child mortality in Brazil: a nationwide longitudinal study of 19 million newborn babies. Lancet Glob Health. (2022) 10(10):e1453–62. 10.1016/S2214-109X(22)00333-3 36113530 PMC9638038

[40] Cardoso AM Santos RV Coimbra CEA Jr. Infant mortality by race/color in Brazil: what do the national information systems say? Cadernos Saúde Pública. (2005) 21:1602–8. 10.1590/S0102-311X2005000500035 16158168

[41] Wong LLR Sánchez JA. The Afro-descendant and Indigenous Population in Latin America: Points of Reflection for the Debate on Cairo +20. Belo Horizonte, BH: Latin American Population Association (ALAP) (2014).

[42] Moreira EAF Oliveira ICDE Andrade FBDE. Infant morbidity and mortality with a focus on perinatal causes in northeastern Brazil. Rev Ciência Plural. (2020) 6(3):1–15. 10.21680/2446-7286.2020v6n3ID19943

[43] Alves TF Fontenelle LF Sarti TD. Mortality from external causes in children and adolescents aged 5 to 14 years. Rev Brasil Pesquisa Saúde. (2022) 24(2):47–54. 10.1590/1413-81232023282.11672022

